# Effect of dietary restriction and subsequent re-alimentation on the transcriptional profile of bovine jejunal epithelium

**DOI:** 10.1371/journal.pone.0194445

**Published:** 2018-03-19

**Authors:** Kate Keogh, Sinead M. Waters, Paul Cormican, Alan K. Kelly, David A. Kenny

**Affiliations:** 1 Animal and Bioscience Research Department, Animal and Grassland Research and Innovation Centre, Teagasc, Grange, Dunsany, Co. Meath, Ireland; 2 School of Agriculture and Food Science, University College Dublin, Belfield, Dublin 4, Ireland; University of Lübeck, GERMANY

## Abstract

Compensatory growth (CG), an accelerated growth phenomenon which occurs following a period of dietary restriction is utilised worldwide in animal production systems as a management practise to lower feed costs. The objective of this study was to evaluate the contribution of jejunal epithelial to CG in cattle through transcriptional profiling following a period of dietary restriction as well as subsequent re-alimentation induced CG. Sixty Holstein Friesian bulls were separated into two groups; RES and ADLIB, with 30 animals in each. RES animals were offered a restricted diet for 125 days (Period 1) followed by *ad libitum* feeding for 55 days (Period 2). ADLIB animals had *ad libitum* access to feed across both periods 1 and 2. At the end of each period, 15 animals from each treatment group were slaughtered, jejunal epithelium collected and RNAseq analysis performed. Animals that were previously diet restricted underwent CG, gaining 1.8 times the rate of their non-restricted counterparts. Twenty-four genes were differentially expressed in RES compared to ADLIB animals at the end of Period 1, with only one gene, *GSTA1*, differentially expressed between the two groups at the end of Period 2. When analysed within treatment (RES, Period 2 v Period 1), 31 genes were differentially expressed between diet restricted and animals undergoing CG. Dietary restriction and subsequent re-alimentation were associated with altered expression of genes involved in digestion and metabolism as well as those involved in cellular division and growth. Compensatory growth was also associated with greater expression of genes involved in cellular protection and detoxification in jejunal epithelium. This study highlights some of the molecular mechanisms regulating the response to dietary restriction and subsequent re-alimentation induced CG in cattle; however the gene expression results suggest that most of the CG in jejunal epithelium had occurred by day 55 of re-alimentation.

## Introduction

In the wild, animals encounter periods of nutrient abundance as well as times of nutrient deficiency. In order to cope with fluctuations in nutrient availability, many animals have evolved the capacity to display accelerated tissue growth and deposition during times of elevated nutrient abundance [1]. Termed compensatory growth (CG), this naturally occurring phenomenon enables animals to undergo enhanced growth and efficiency upon re-alimentation following a prior dietary restriction [2]. The occurrence of CG has been incorporated into many livestock production systems, particularly for cattle as a method to reduce feed input costs [2]. However, although this naturally occurring phenomenon is utilised worldwide [3–6], there is a dearth of knowledge in relation to the molecular control regulating the expression of the trait in cattle. Previous molecular based analyses of this trait, by our own group in muscle, liver and ruminal papillae tissues have indicated alterations in the expression of genes involved in processes including metabolism, cellular division and growth and cellular organisation during CG [7–9]. However further investigations into the molecular expression of this trait in other metabolically important tissues is warranted, as a greater understanding of the control of CG at the molecular level would lead to better exploitation and possible incorporation of this economically important trait into genomic selection breeding programs for beef cattle.

Organs including components of the gastrointestinal tract have repeatedly been shown to display accelerated growth upon re-alimentation following a prior dietary restriction [3–6].

Indeed, metabolic organs including the gastrointestinal tract and liver typically display the initial greatest growth rates and can compensate before other tissues or organs in the body [10] which may be regarded as a direct response to increased metabolic activity as a consequence of increased dietary intake [6]. Moreover, a number of studies have noted physical alterations to intestinal epithelium following dietary restriction and subsequent re-alimentation induced CG in rodents [11–12]; fish [13] and reptiles [14] as well as in livestock species such as goats [15] and pigs [16]. In these studies small intestinal atrophy and structural changes, including the disappearance of villi and a reduction in the size and number of crypts, was evident in response to both moderate and severe dietary restriction. However, during subsequent re-alimentation and associated CG, an increase in intestinal surface area and restoration of intestinal epithelium was apparent. Moreover, the small intestine which plays a central role in starch utilisation and nutrient absorption has been shown to adapt to altering planes of nutrition in cattle through modifying tissue form and function [17]. Therefore, the objective of this study was to examine the transcriptional profile of the jejunum, which is of primary importance as a site of digestion and also in the absorption of nutrients through the intestinal wall, in response to a period of dietary restriction and also a period of re-alimentation induced CG. During CG our attention was focused on the first 55 days of re-alimentation in order to capture the maximal accelerated growth of re-alimentation [2]. Additionally, during CG animals are typically more feed efficient, thus a secondary objective was to evaluate the contribution of jejunal epithelial to the improved feed efficiency apparent during CG.

## Materials and methods

The University College Dublin Animal Research Ethics Committee approved all procedure using animals and the current study was licensed by the Irish Department of Health and Children in accordance with the European Community Directive 86/609/EC.

### Animal management

This experiment was performed as a component of a larger research programme aimed at describing the effect of dietary restriction and subsequent re-alimentation on overall body physiology [6, 18]. Details of the management of animals used are outlined in full in Keogh et al. [6, 18] and are only briefly described here. Sixty Holstein Friesian bulls (mean (SEM) age: 479 (15) days; bodyweight 370 (35) kg) were separated into two groups; RES and ADLIB, with 30 animals in each. RES animals were offered a restricted diet for 125 days (Period 1) followed by *ad libitum* access to feed for 55 days (Period 2). ADLIB animals had *ad libitum* access to feed across both periods 1 and 2. All animals received the same diet consisting of 70:30 concentrate:forage (grass silage) throughout the entire trial, but with a differing proportion based on treatment group. The concentrate ration consisted of rolled barley (72.5%), soyabean meal (22.5%), molasses (3%) and mineral supplement (2%). Additionally all animals were individually fed, with the proportion of feed offered based on individual bodyweight and animals were weighed regularly throughout the trial. On average, RES animals consumed 57% less feed than the ADLIB group during Period 1. During the dietary restriction phase (Period 1) RES animals were managed to grow at 0.6 kg/day. At the end of each period 15 animals from each group, RES and ADLIB were slaughtered.

### Tissue sampling

All animals were humanely slaughtered in an EU licensed abattoir (Euro Farm Foods Ltd, Cooksgrove, Duleek, Co. Meath, Ireland) through captive bolt stunning followed by exsanguination and all tissue samples were harvested post slaughter. Jejunal tissue (10 cm) was harvested approximately 30 cm distal to the duodenal-jejunal juncture. Samples were collected and placed in Dulbecco phosphate buffered saline (DPBS) to remove any digesta. Jejunum sections were initially washed in DPBS and subsequently cut along the longitudinal axis to allow the tissue to be laid flat. Following opening of the tissue, jejunum epithelium samples were washed for a second time in DPBS to ensure that no digesta remained on the tissue. Epithelial tissue was then scraped from the underlying connective and muscular tissue using a glass microscope slide. The tissue was then placed in a collection tube snap frozen in liquid nitrogen and subsequently stored at -80°C.

### RNA isolation, sequencing and bioinformatics analysis

RNA isolation, cDNA library preparation and sequencing as well as bioinformatic analysis have been outlined previously [8, 9] and are only briefly described here. Total RNA was isolated from approximately 30 mg of frozen jejunal epithelium using an RNeasy Mini Kit (Qiagen, UK), according to the manufacturer’s instructions. The quantity of RNA and RNA integrity were determined using a Nanodrop spectrophotometer ND-1000 (Nanodrop Technologies, Wilmington, DE, USA) and the RNA 6000 Nano Lab Chip kit (Agilent Technologies Ireland Ltd., Dublin, Ireland), respectively. Only high quality RNA samples (RNA integrity numbers >8) were selected for subsequent RNA sequencing (10 samples from each treatment group at each slaughter time-point). cDNA libraries were prepared from 3 μg of high quality total RNA using the Illumina TruSeq RNA sample prep kit following the manufacturer’s instructions (Illumina, San Diego, CA, USA). In total, 40 individual RNAseq libraries were multiplexed according to their respective sample specific adapters and 100 base-pair single end sequencing was performed across 4 flowcell lanes on an Illumina HiSeq 2000 sequencer.

Raw sequence reads were first checked for quality using FASTQC software (version 0.10.0) and were then trimmed of low quality reads using Trim Galore. Trimmed reads were then aligned to the bovine reference genome (UMD3.1) using TopHat (v2.0.9) and HTSeq (v0.5.4p5) (http://pypi.python.org/pypi/HTSeq) was used to calculate the number of sequence reads aligned to all protein-coding genes from the ENSEMBL v74 annotation of the bovine genome. EdgeR (v3.4.1), was then used to identify statistically significant (P<0.05) differentially expressed genes (DEGs), through a generalised linear model likelihood ratio test. The following treatment comparisons were tested for DEGs: (i) RES *v*. ADLIB at the end of Period 1; (ii) RES *v*. ADLIB at the end of Period 2; (iii) RES Period 2 *v*. RES Period 1; and (iv) ADLIB Period 2 v. ADLIB Period 1. Statistically significant (P<0.05) DEGs with a Benjamini-Hochberg false discovery rate of < 0.1% were deemed to be significant. Pathway and functional analyses of DEGs were then undertaken using Ingenuity Pathway Analysis (IPA; v. 8.8, Ingenuity Systems, Mountain View, CA; http://www.ingenuity.com).

## Results

### Animal performance

The effect of dietary regimen on body-weight gain, feed intake and animal performance are outlined in detail by Keogh et al. [6]. Briefly, following 125 days of differential feeding at the end of Period 1, RES animals were 161 kg lighter than ADLIB animals (RES: 442 v ADLIB: 603 kg, respectively). A period of 55 days of *ad libitum* feeding for both groups in Period 2, resulted in a reduction in the body weight difference between treatment groups (84 kg difference; 594 and 678 kg for RES and ADLIB, respectively). Overall, animals undergoing re-alimentation induced CG compensated for 48% of their previous under-performance in only 55 days of re-alimentation. During Period 1 body-weight gain for RES animals was 0.6 kg/day, whilst ADLIB animals gained 1.9 kg/day during the same time. Following a period of re-alimentation in Period 2, RES animals gained 2.5 kg/day with ADLIB animals growing at 1.4 kg/day. RES animals had a lower overall dietary intake during Period 1; however, during Period 2 there was no difference in intake between treatment groups. As a consequence, feed efficiency index, feed conversion ratio was enhanced in RES during re-alimentation in Period 2 (4.87) compared to RES Period 1 and ADLIB animals across both periods (Period 1: RES: 9.5; ADLIB: 6.71; Period 2: ADLIB: 9.98).

### mRNA read alignment and differential gene expression

Approximately 83% of RNAseq reads aligned to the bovine genome, and approximately 70% of those that were aligned, were mapped to protein coding genes. At the end of Period 1, 13,685 genes were expressed with 13,605 genes expressed at the end of Period 2. Following a period of dietary restriction, at the end of Period 1, 24 genes were identified as differentially expressed in RES compared to ADLIB animals. However, following 55 days of subsequent re-alimentation only one gene, *GSTA1* (*P* < 0.001; fold change: 6.94) was differentially expressed between treatment groups. When the data were analysed within treatment (RES, Period 2 v Period 1), 31 genes were observed to be differentially expressed in animals undergoing CG compared to their contemporaries during earlier dietary restriction. Only one gene was identified as differentially expressed within the ADLIB group between Period 1 and 2; *ANPEP* was down-regulated in ADLIB Period 2 compared to ADLIB Period 1 (*P* < 0.001; fold change: 4.14). RNAseq data from the current study are available on NCBI’s Gene Expression Omnibus [19] through GEO Series accession number GSE94004.

### Pathway analysis

**Fig 1:**
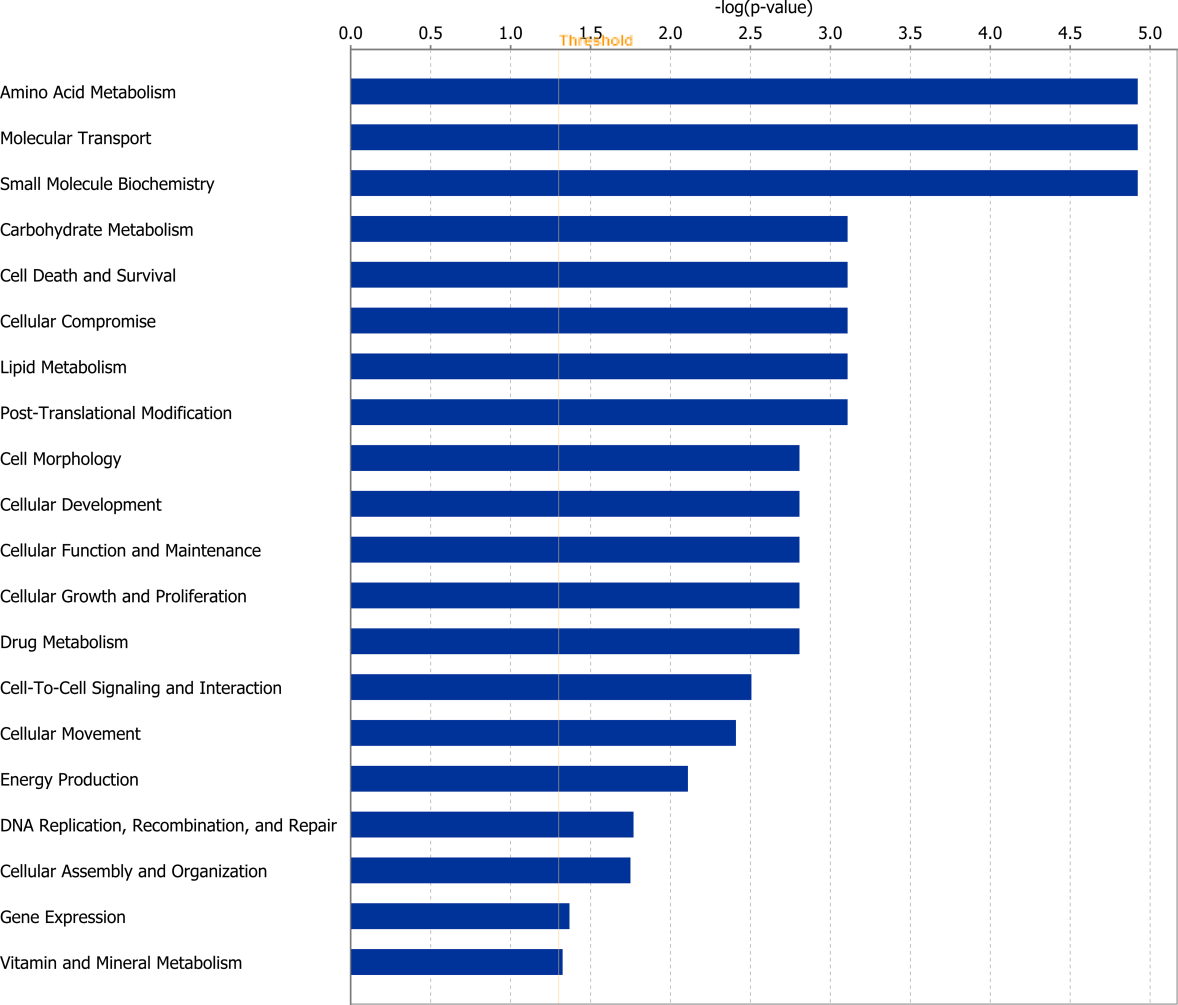
Differentially expressed genes as a consequence of dietary restriction (RES v ADLIB at the end of Period 1) classified according to molecular and cellular function. The bars indicate the likelihood [-log (*P* value)] that the specific function was affected by dietary restriction compared with others represented in the list of differentially expressed genes.

**Fig 2:**
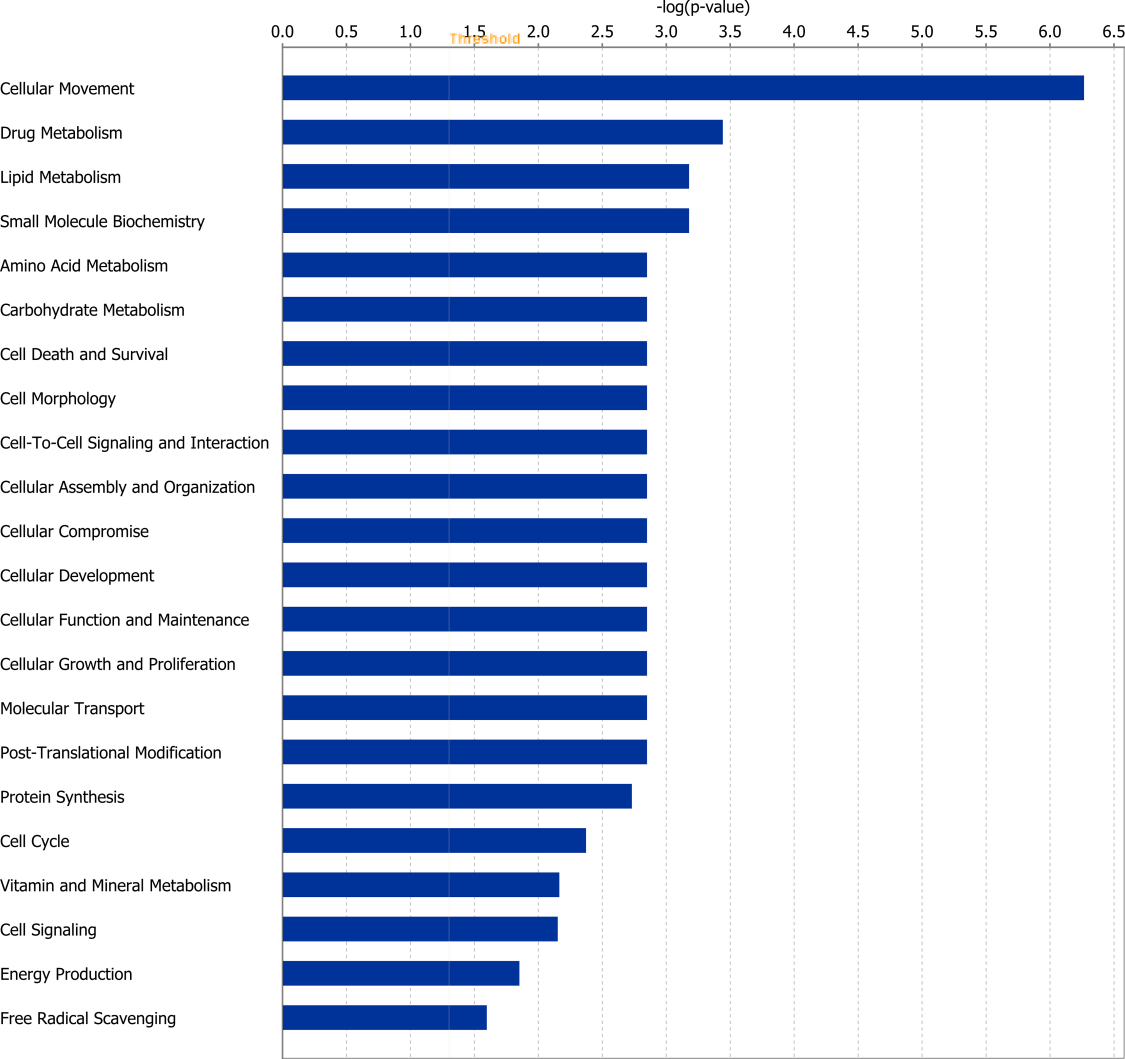
Differentially expressed genes as a consequence of compensatory growth (RES Period 1 v RES Period 2) classified according to molecular and cellular function. The bars indicate the likelihood [-log (*P* value)] that the specific function was affected by dietary restriction compared with others represented in the list of differentially expressed genes.

Of the 24 DEGs at the end of Period 1, 18 genes were successfully mapped to a molecular or biological pathway and/or category in the IPA database. Fold changes of genes identified as differentially expressed at the end of Period 1, between RES and ADLIB animals are presented in Table 1. When analysed within the RES treatment (Period 2 v Period 1), of the 31 genes differentially expressed 30 genes were successfully mapped to a molecular or biological pathway and/or category in the IPA database. Differentially expressed genes within the RES treatment group are outlined in Table 2. DEGs for each comparison were analysed and assigned to particular biological functions within IPA. Within the RES treatment, when gene lists for Period 2 were compared with Period 1, genes involved in processes including amino acid, lipid and carbohydrate metabolism, as well as cellular growth and proliferation and cellular survival were all found to be differentially expressed (*P* < 0.05). Biological categories identified within IPA at the end of Period 1 between RES and ADLIB treatments are presented in Fig 1, with biological categories identified at the end of Period 2 relative to the end of Period 1 in RES animals presented in Fig 2. Specific genes pertaining to each biological category for Figs 1 and 2 are outlined in S1 Table and S2 Table respectively.

**Table 1:** Genes differentially expressed in jejunal epithelial following a period of dietary restriction at the end of Period 1.

Gene ID	Gene name	Fold change^1^
*ANPEP*	Alanyl (membrane) aminopeptidase	-26.2
*ANXA10*	Annexin A10	-4.4
*AP3B2*	Adaptor-related protein complex 3, beta 2 subunit	-8.7
*ASIC3*	Acid sensing (proton gated) ion channel 3	-11.3
*ASNS*	Asparagine synthetase (glutamine-hydrolyzing)	2.3
*CARS*	Cysteinyl-tRNA synthetase	1.6
*CTSW*	Cathepsin W	1.9
*DAPL1*	Death associated protein-like 1	-6.7
*ELL3*	Elongation factor RNA polymerase II-like 3	-3.9
*INSIG1*	Insulin induced gene 1	-1.9
*OLFML3*	Olfactomedin-like 3	2.0
*PAPSS2*	3'-phosphoadenosine 5'-phosphosulfate synthase 2	-3.5
*PGA3*	Pepsinogen-3	-2.8
*S100A2*	S100 calcium binding protein A2	-2.8
*SDS*	Serine dehydratase	-3.1
*SLC1A5*	Solute carrier family 1 (neutral amino acid transporter), member 5	2.0
*SLC7A5*	Solute carrier family 7 (amino acid transporter light chain, L system), member 5	2.1
*WNT2*	Wingless-type MMTV integration site family member 2	2.2

^1^ Fold changes are up or down in restricted fed animals compared to *ad libitum* fed control animals

**Table 2:** Genes differentially expressed in jejunal epithelial following a period of re-alimentation induced compensatory growth (Period 2) relative to following a period of dietary restriction (Period 1).

Gene ID	Gene name	Fold change^1^
*ADIRF*	Adipogenesis regulatory factor	-3.2
*ANXA10*	Annexin A10	3.9
*ASNS*	Asparagine synthetase (glutamine-hydrolyzing)	-2.4
*CMA1*	Chymase 1, mast cell	-3.1
*DAPL1*	Death associated protein-like 1	6.6
*DDAH1*	Dimethylarginine dimethylaminohydrolase 1	2.3
*DNAH2*	Dynein, axonemal, heavy chain 2	2.6
*EFR3B*	EFR3 homolog B (S. cerevisiae)	2.3
*GCNT3*	Glucosaminyl (N-acetyl) transferase 3, mucin type	8.9
*GSTA1*	Glutathione S-transferase alpha 1	16.7
*HERPUD1*	Homocysteine-inducible, endoplasmic reticulum stress-inducible, ubiquitin-like domain member 1	-1.7
*IL17RB*	Interleukin 17 receptor B	-1.9
*INSIG1*	Insulin induced gene 1	2.4
*IRG1*	Immunoresponsive 1 homolog (mouse)	3.0
*LRRC17*	Leucine rich repeat containing 17	-2.9
*LTC4S*	Leukotriene C4 synthase	-2.3
*LTF*	Lactotransferrin	2.9
*MAP1LC3C*	Microtubule-associated protein 1 light chain 3 gamma	-2.9
*PFKFB3*	6-phosphofructo-2-kinase/fructose-2,6-biphosphatase 3	2.2
*PGA3*	Pepsinogen-3	4.9
*PLP1*	Proteolipid protein 1	-2.8
*PRLR*	Prolactin receptor	2.1
*PSAT1*	Phosphoserine aminotransferase 1	-2.6
*S100A2*	S100 calcium binding protein A2	5.1
*SCG2*	Secretogranin II	-3.2
*SDS*	Serine dehydratase	4.8
*SDSL*	Serine dehydratase-like	3.1
*SLAMF7*	SLAM family member 7	-1.9
*TFF2*	Trefoil factor 2	18
*TNFRSF11B*	Tumor necrosis factor receptor superfamily, member 11b	2.1

^1^Fold changes are within the RES treatment group in compensating animals compared with restricted fed animals.

## Discussion

The accelerated growth phenomenon that is CG is widely known to occur in ruminant species and indeed is utilised in many production settings in order to reduce feed costs [2, 4, 5, 20]. The gastrointestinal tract has previously been shown to be one of the most responsive organs to both dietary restriction and subsequent CG [3–6], thus our attention was focused on examining the transcriptional profile of the jejunum, which is of primary importance as a site of digestion and also in the absorption of nutrients through the intestinal wall. The objective of the current study was to evaluate the transcriptional response of jejunal epithelial to the expression of CG. This was achieved through an examination of DEGs in jejunal epithelial following a period of dietary restriction and also a period of subsequent re-alimentation compared to that of animals that were fed continuously. Additionally, in order to further assess the effect of CG on the jejunal transcriptome, sequencing data were analysed within treatment group. When analysed within treatment across time, the larger difference in DEGs between RES and ADLIB groups (RES: 31 DEGs, ADLIB: 1 DEG) suggests that the RES treatment group over time analysis is reflective of CG and not of normal growth as described in the ADLIB DEG profile.

### Digestion and metabolism

We observed DEGs coding for proteins involved in digestion and metabolism following a period of dietary restriction as well as during CG. As the jejunum comprises a key component of the gastrointestinal tract with important roles in digestion and absorption of nutrients, alterations in the expression of such genes was not entirely unexpected. Indeed previous investigations into the effect of dietary restriction and subsequent re-alimentation in another primary metabolic tissue, the liver, have also reported alterations in the expression of genes associated with protein, lipid and carbohydrate metabolism [8, 21]. At the end of Period 1 in the current jejunal epithelial study, two genes, *ANPEP* and *PGA3*, which are both involved in digestive processes were down-regulated in RES animals compared to ADLIB animals. In the small intestine the aminopeptidase, ANPEP, functions in the final digestion of peptides generated from hydrolysis of proteins by gastric and pancreatic proteases [22]. Similarly this gene was also down-regulated in hepatic tissue in the same animals used in the current study [8]. *PGA3* codes for the inactive precursor of pepsinogen, which is released by the gastric chief cells in the stomach and functions in the further degradation of food into peptides [23]. During the process of digestion, these enzymes, each of which is specialised in severing links between particular types of amino acids, collaborate to break down dietary proteins into their components, which can be readily absorbed by the intestinal lining [24]. Down-regulation of both of these digestive genes suggests a lower requirement for digestive processes in jejunal epithelial of RES animals during Period 1, which may be reflective of the lowered feed intake of these animals during that time [6].

As mentioned previously, dietary restriction and subsequent re-alimentation have been shown to cause differential expression of genes involved in metabolism [8, 21]. Indeed, alterations in the expression of metabolism genes were also evident in jejunal epithelium in the current study. Following a period of differential feeding, lower expression of *INSIG1* and *SDS* which encode proteins involved in regulating cholesterol biosynthesis and serine and glycine metabolism respectively [25, 26] was evident in RES compared to ADLIB animals. *INSIG1* expression was also reported to be down-regulated in skeletal muscle tissue of cattle undergoing a period of dietary restriction [7]. Lower systemic glucose and consequently insulin following dietary restriction is well described for cattle [18, 27–29]. Consequently, as insulin concentrations regulate *INSIG1* expression, down-regulation of this gene during dietary restriction may have been reflective of the lowered systemic glucose and insulin concentrations observed [18]. The effect of insulin concentrations on this gene in relation to dietary restriction and CG are further established through up-regulation of this gene in hepatic tissue during re-alimentation induced CG [8, 21]. The primary role of *SDS* is in the metabolism of serine and glycine, concomitant with the production of pyruvate [26]. *SDS* has previously been reported to be affected by dietary restriction and subsequent re-alimentation, with lower and greater expression of this gene evident in skeletal muscle following a period of dietary restriction and subsequent re-alimentation, respectively [7]. Again down-regulation of these genes may have reflected a lowered requirement for metabolic processes in this tissue in response to a restricted dietary regimen.

In addition to its functionality in digestive and metabolic processes the jejunum is also a primary site for the absorption of digested nutrients across the intestinal wall for uptake and further metabolism in the liver [30]. At the end of Period 1, up-regulation of two genes coding for solute-like carrier amino acid transporters, namely *SLC1A5* and *SLC7A5* was apparent. The jejunum has previously been identified as the major site of amino acid and peptide absorption within the small intestine [30, 31]. The greater expression of *SLC1A5* and *SLC7A5* observed in the current study may reflect an enhanced requirement for the uptake of amino acids and a greater utilisation of diet derived nutrients during dietary restriction.

In the current study a period of dietary restriction was associated with down-regulation of genes involved in metabolism and digestion. Conversely, however, during re-alimentation DEGs involved in metabolism and digestion were subsequently up-regulated. For example during re-alimentation genes involved in metabolism including *PGA3*, *PFKB3*, *SDS* and *SDSL* were up-regulated in animals undergoing CG relative to that observed during dietary restriction (RES Period 2 compared to RES Period 1). *PFKFB3* codes for an enzyme involved in glycolysis [32], whereas *SDS* and *SDSL* both encode genes involved in serine and glycine metabolism. Consistent with this, Connor et al. [21] and Keogh et al. [8] both observed greater expression of genes involved in metabolism during re-alimentation induced CG in hepatic tissue. Greater expression of metabolism genes during Period 2 occurred with a greater dietary intake in the animals undergoing re-alimentation induced CG [6] which may have reflected a greater requirement for metabolic processes concomitant with greater dietary intake in jejunal epithelial during this time. However, further studies are required to assess the metabolic state of the metabolic organs in response to both dietary restriction and CG.

### Cellular growth and differentiation

Intestinal villi have previously been shown to be responsive to plane of nutrition, with alterations in villi size apparent under conditions of nutrient restriction as well as in response to subsequent re-alimentation [16, 33]. Indeed, Sun et al. [15] observed that jejunal villus height and width were smaller in goats that had been fed a restricted diet for 48 days, compared to those that had not been restricted. Following a subsequent period of re-alimentation induced CG (63 days) there was no difference in jejunal villus height or width between treatment groups [15], further establishing the role of intestinal morphology in response to plane of nutrition. Similarly in the current study, although physical alterations in intestinal villi were not assessed, down-regulation of genes involved in cellular growth and differentiation was apparent following a period of dietary restriction. Effects of dietary restriction and subsequent re-alimentation on the expression of genes involved in cellular growth and differentiation have previously been reported in the literature, namely in hepatic tissue [8, 21] and also skeletal muscle tissue [34, 35]. In the current study with jejunal epithelial, *ANXA10*, which encodes a member of the annexin family which are involved in the regulation of cellular growth [36]; *DAPL1*, which is involved in the early stages of epithelial differentiation [37]; and *S100A2* which encodes a member of the S100 family of proteins which are involved in the regulation of both cell cycle progression and differentiation [38, 39] were all down-regulated at the end of Period 1 in RES animals compared to ADLIB animals. Down-regulation of these genes at the end of Period 1 suggests a reduction in cellular growth or division processes in jejunal epithelial following a period of differential feeding. Moreover, a SNP in the *ANXA10* gene has previously been shown to be associated with feed efficiency in Nellore cattle [40]. Additionally, in the current study, greater expression of *ASNS* was also apparent at the end of Period 1. This gene has previously been shown to be capable of blocking progression through the G1 phase of the cell cycle and inhibiting cellular proliferation [41]. Thus, greater expression of this gene implies an inhibition of cellular division following a period of dietary restriction in RES animals at the end of Period 1. Greater expression of *ASNS* was also apparent in mice following a period of protein restriction [42], as well as in the hepatic tissue of the same cattle used here during dietary restriction [8]. Moreover, differential expression of *ASNS* was also evident in skeletal muscle tissue in response to both dietary restriction and subsequent re-alimentation in the data of Keogh et al. [7]. Lower expression of the transcriptional elongation factor, *ELL3* was also evident in jejunal epithelia at the end of Period 1. Elongation factors function to increase the catalytic rate of RNA polymerase II transcription by suppressing transient pausing by the polymerase at multiple sites along the DNA strand [43]. These apparent alterations to growth and cellular division processes in jejunal epithelium may be due to alterations in the overall metabolic activity or workload within the gastrointestinal tract as a consequence of a lowered nutrient intake. Alternatively, such reductions in cellular growth and division may be due to a requirement to maintain cellular metabolic homeostasis rather than direct diet derived nutrient energy intake towards cellular growth and proliferation during a period of dietary restriction.

During re-alimentation, up-regulated growth processes in jejunal epithelium may have contributed to CG in these cells. This was apparent through the subsequent up-regulation of *S100A2* and *LTF*, which function in the regulation of cellular growth and differentiation [44] during re-alimentation. Greater expression of genes involved in cellular division and proliferative processes has also previously been identified as a contributory factor towards the expression of CG in cattle [8, 21]. Indeed, more specifically, greater expression of *S100A2* was also apparent in skeletal muscle tissue during re-alimentation induced CG [7]. Moreover, Levesque et al. [16] and Sun et al. [15] both reported an increase in jejunal villus height during re-alimentation induced CG in pigs and goats, respectively, further underpinning greater cellular growth and proliferation in intestinal tissue during CG. Indeed, Levesque et al. [16] postulated that the increase in villus height during re-alimentation may allow for an improvement in nutrient digestibility and ultimately may be contributing to the occurrence of CG through an improvement in digestive capability. Additionally, greater numbers of villi or increased villi height may lead to greater surface area which would increase absorptive capacity of the jejunum, ultimately contributing to greater utilisation of dietary intake and improved feed efficiency which is characteristic of CG.

### Immune function and cellular detoxification

Reports in the literature on calorie restriction in species including mice, rats and humans have described effects on the immune system, most notably an improved immune function following a period of dietary restriction [45–49]. Moderate dietary restriction can affect survival rates of laboratory animals by reducing cellular division and delaying the aging process, which can consequently affect the immune system [50]. Indeed, Pahlavani [50] reported a superior immunological status in rodents that had been offered a restricted diet compared to that of non-restricted rodents. This effect of diet restriction on the immune system may lead to a more active and prominent immune response for the organism which may be advantageous should there be any threat of pathogens or infections to the organism. Indeed in the current study, at the end of a period of dietary restriction, *CTSW*, which codes for a cysteine proteinase that functions in regulating T-cell cytolytic activity [51] was up-regulated in RES compared to ADLIB animals. However more prominent evidence for an effect of dietary restriction on the immune system was apparent through genes differentially expressed in animals undergoing re-alimentation induced CG relative to those at the end of a period of dietary restriction. In this comparison, differential expression of immune related genes included up-regulation of *IRG1* and down-regulation of *IL17RB*, *LTC4S*, *MAP1LC3C*, *SLAM7* and *CMA1* which was evident in cattle undergoing re-alimentation induced CG compared to those fed restrictedly at the end of Period 1 (RES Period 2 relative to RES Period 1). Immune genes identified as differentially expressed reflected different types of immune response including inflammation and autophagy. For example, *IRG1* codes for a protein involved in the inhibition of the inflammatory response, and acts as a negative regulator of the Toll-like receptor-mediated inflammatory response [52]. *IL17RB* codes for a cytokine receptor [53]. *LTC4S* codes for a mediator of inflammation [54]. *SLAMF7* belongs to a family of signalling lymphocytic activation molecule receptors which are cell specific receptors with critical roles in normal immune regulation [55]. *MAP1LC3C* codes for a protein that plays a role in antibacterial autophagy [56], and *CMA1* codes for a serine proteinase which is expressed in mast cells and functions in the degradation of the extracellular matrix [57]. Elsasser et al. [58] suggested that the immune system may be involved in nutrient partitioning with up-regulation of immune genes causing activation of tissue mobilisation during dietary restriction with the corollary resulting in more energy to be partitioned towards growth during periods of greater dietary consumption. Thus, down-regulation of immune-related genes during CG may be an inherent adaptation in response to re-alimentation in order to allow more energy to be partitioned towards growth, as suggested by Elsasser et al. [58]. Overall, these results suggest that dietary restriction in cattle can elicit a superior immunological status as previously described in other species which may prevent any potential pathological threats to the animal as well as potentially allowing for more dietary derived energy to be partitioned towards growth during re-alimentation.

At the end of Period 2, only one gene was differentially expressed between RES and ADLIB animals, namely *GSTA1*. This gene codes for a glutathione S-tranferase which is involved in cellular detoxification and was up-regulated in RES relative to ADLIB animals following a period of re-alimentation. Additionally, *TFF2* and *DDAH1* were also up-regulated in jejunal tissue of cattle undergoing CG (RES Period 2 relative to RES Period 1). *TFF2* codes for a protein involved in the protection of the intestinal mucosa [59] whilst *DDAH1* and *GSTA1* both function in cellular detoxification [60, 61]. Up-regulation of genes coding for functions such as detoxification and cellular protection suggests a greater requirement for detoxification during CG and greater feed intake. Greater expression of glutathione s-transferase genes has been reported previously during re-alimentation following a prior dietary restriction, in skeletal muscle tissue (*GSTK1*, [7]) and hepatic tissue (*GSTA1*, *GSTZ1*, *GSTM4*, [21]). A similar response was also reported in skeletal muscle where during early re-alimentation greater expression of genes coding for FoxO proteins was evident [62]. Up-regulation of genes involved in cellular protection and detoxification may be an acquired adaptive response to increased nutrient intake during re-alimentation. For example a sudden increase in nutrient intake may lead to an associated increase in the rate of oxidative metabolism, which may in turn result in the production of reactive oxygen species, potentially detrimental to cellular survival [63]. Thus, there is potential that up-regulation of genes associated with protective or detoxification roles may be necessary to preserve a homeostatic state within the jejunal epithelium during periods of greater feed intake. This has also been reported during instances of greater states of cellular nutrient abundance and associated stress *in vitro* in mammalian cells [64]. Overall, differential expression of genes involved in immunity and cellular detoxification suggest a greater immune response during dietary restriction and a subsequent requirement to maintain cellular homeostasis and survival during subsequent re-alimentation and CG.

## Conclusions

Following a period of dietary restriction, genes associated with metabolism and digestion were down-regulated in response to reduced dietary intake. However, subsequent to this when these cattle were undergoing re-alimentation and CG; genes associated with these processes were observed to be up-regulated. Indeed, greater nutrient intake during re-alimentation was also associated with increased expression of genes involved in cellular protection and detoxification. Reduced and then subsequently increased dietary intake and resultant gastrointestinal processing may have led to alterations in jejunal villi numbers or structure. We observed evidence for this in the current study through lower expression of genes involved in growth and cellular division following a period of dietary restriction with the opposite effect evident during re-alimentation. Indeed greater numbers of jejunal villi may result in an increase in the surface area for absorption and thus facilitate an increase in feed efficiency, which is typically observed in animals undergoing CG. Finally, results from this study suggest that a moderate dietary restriction and subsequent CG may affect the immune response, which may reflect an acquired adaptive response in order to cope with changes in nutrient abundance and associated tissue mobilisation and deposition. This study provides an insight into the contribution of the jejunum, a key segment of the digestive machinery of the gastrointestinal tract. However, given that only one gene was differentially expressed by day 55 of re-alimentation, we must conclude that differential gene expression in this tissue is unlikely to contribute long-term to the CG phenomenon in cattle.

## Supporting information

S1 Table Genes pertaining to biological categories identified from Fig 1 from differentially expressed genes between RES and ADLIB treatment groups at the end of Period 1. (XLS)

S2 Table Genes pertaining to biological categories identified from Fig 2 from differentially expressed genes within RES animals between re-alimentation in Period 2 and dietary restriction in Period 1. (XLSX)
